# *In vitro* biomechanical properties, fluorescence imaging, surface-enhanced Raman spectroscopy, and photothermal therapy evaluation of luminescent functionalized CaMoO_4_:Eu@Au hybrid nanorods on human lung adenocarcinoma epithelial cells

**DOI:** 10.1080/14686996.2016.1189797

**Published:** 2016-07-26

**Authors:** Qifei Li, Abdul K. Parchur, Anhong Zhou

**Affiliations:** ^a^ Department of Biological Engineering, Utah State University, Logan, UT, USA

**Keywords:** Luminescent functionalized hybrid nanoparticles, biomechanical properties, surface-enhanced Raman spectroscopy (SERS), photothermal therapy (PTT), multi-modal imaging probe, 30 Bio-inspired and biomedical materials, 102 Porous/Nanoporous/Nanostructured materials, 212 Surface and interfaces, 204 Optics/Optical applications

## Abstract

Highly dispersible Eu^3+^-doped CaMoO_4_@Au-nanorod hybrid nanoparticles (HNPs) exhibit optical properties, such as plasmon resonances in the near-infrared region at 790 nm and luminescence at 615 nm, offering multimodal capabilities: fluorescence imaging, surface-enhanced Raman spectroscopy (SERS) detection and photothermal therapy (PTT). HNPs were conjugated with a Raman reporter (4-mercaptobenzoic acid), showing a desired SERS signal (enhancement factor 5.0 × 10^5^). The HNPs have a heat conversion efficiency of 25.6%, and a hyperthermia temperature of 42°C could be achieved by adjusting either concentration of HNPs, or laser power, or irradiation time. HNPs were modified with antibody specific to cancer biomarker epidermal growth factor receptor, then applied to human lung cancer (A549) and mouse hepatocyte cells (AML12), and *in vitro* PTT effect was studied. In addition, the biomechanical properties of A549 cells were quantified using atomic force microscopy. This study shows the potential applications of these HNPs in fluorescence imaging, SERS detection, and PTT with good photostability and biocompatibility.

## Graphical abstract

**Figure:**
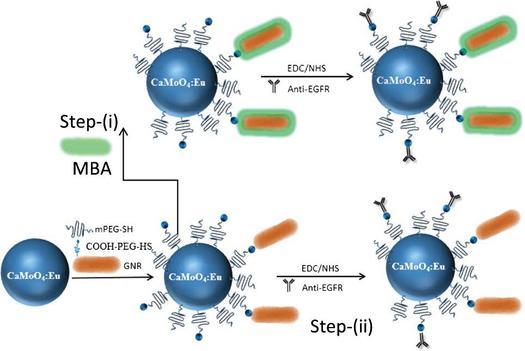

## 1. Introduction

Development of novel nanostructured materials with luminescent, surface-enhanced Raman spectroscopy (SERS) and photothermal therapy (PTT) properties has drawn significant interest in clinical diagnosis and therapeutic monitoring in biological systems.[1–5] Ultrasensitive and non-invasive detection of specific bioanalytes in living cells can be achieved by SERS though increasing the weak inelastic scattering signal into a structurally sensitive probe.[6] To realize this SERS function with good stability and biocompatibility, gold nanorods (GNRs) are conjugated with Raman reporter molecules followed by protective polymers (e.g. polyethylene glycol, PEG).[7] PTT reagents such as GNRs absorb near-infrared (NIR) photons and convert them into heat energy (hyperthermia temperature 42°C) to destroy the cancer cells.[8] Hybrid nanoparticles (HNPs) exhibiting fluorescence emission (615 nm), good photothermal stability, and high biocompatibility are potential candidates for cancer therapy. Also, GNRs have high tissue penetration in the NIR region (700–850 nm). HNPs have been used as a photothermal therapy contrast where the heating effect is induced by the GNRs, which can be monitored using the emission spectrum.[9–11] These types of particles can be efficiently used for diagnosis and selective PTT of cancer cells. Recently, PTT agents such as GNRs,[12] Au nanoshells,[13] zinc ferrite spinel reduced graphene oxide (ZnFe_2_O_4_–rGO),[14] palladium nanostructures,[15] CuS nanoparticles (NPs),[16] Cu_9_S_5_ nanocrystals,[17] and other inorganic NPs have been intensively investigated. However, none of these nanostructured materials have fluorescence properties. It is also known that the proximity of GNRs on the surface of luminescent NPs (e.g. lanthanide ion doped NPs or dye molecules) significantly enhances the luminescence efficiency or quenches their emission.[11,18] HNPs can be used for both *in vivo* fluorescence imaging and PTT.

Cellular biomechanics (Young’s modulus and adhesion) can be considered as an indicator of early diagnosis of cancers, where cancer cells have lower biomechanics (e.g. lower cellular stiffness) than their normal counterparts.[19] When the NPs interact with cells, the proteins present in the cell membrane bind to the surface of NPs and form a coating known as the protein corona. Rapid corona formation affects NPs uptake and the death of endothelial cell at an early stage.[20] Although many NPs for therapeutic applications have been studied, little is known about the morphological and biomechanical changes of cancer cells induced by NPs. Moreover, attaining specific targeting of NPs in a tumor site is particularly important. This can be achieved by conjugating antibodies (Ab) to the HNPs. Epidermal growth factor receptor (EGFR), one of the cell surface biomarkers for targeting in Ab-based cancer therapy, is a transmembrane receptor protein embedded in the plasma membrane of many types of cells. Overexpression of EGFR (>50%) is observed in lung cancer patients.[21,22] Recent studies have shown that NPs labeled with anti-EGFR Ab could effectively kill the target cancer cells when irradiated by laser light with a wavelength around the nanoparticle absorption peak.[23,24]

**Figure 1.**
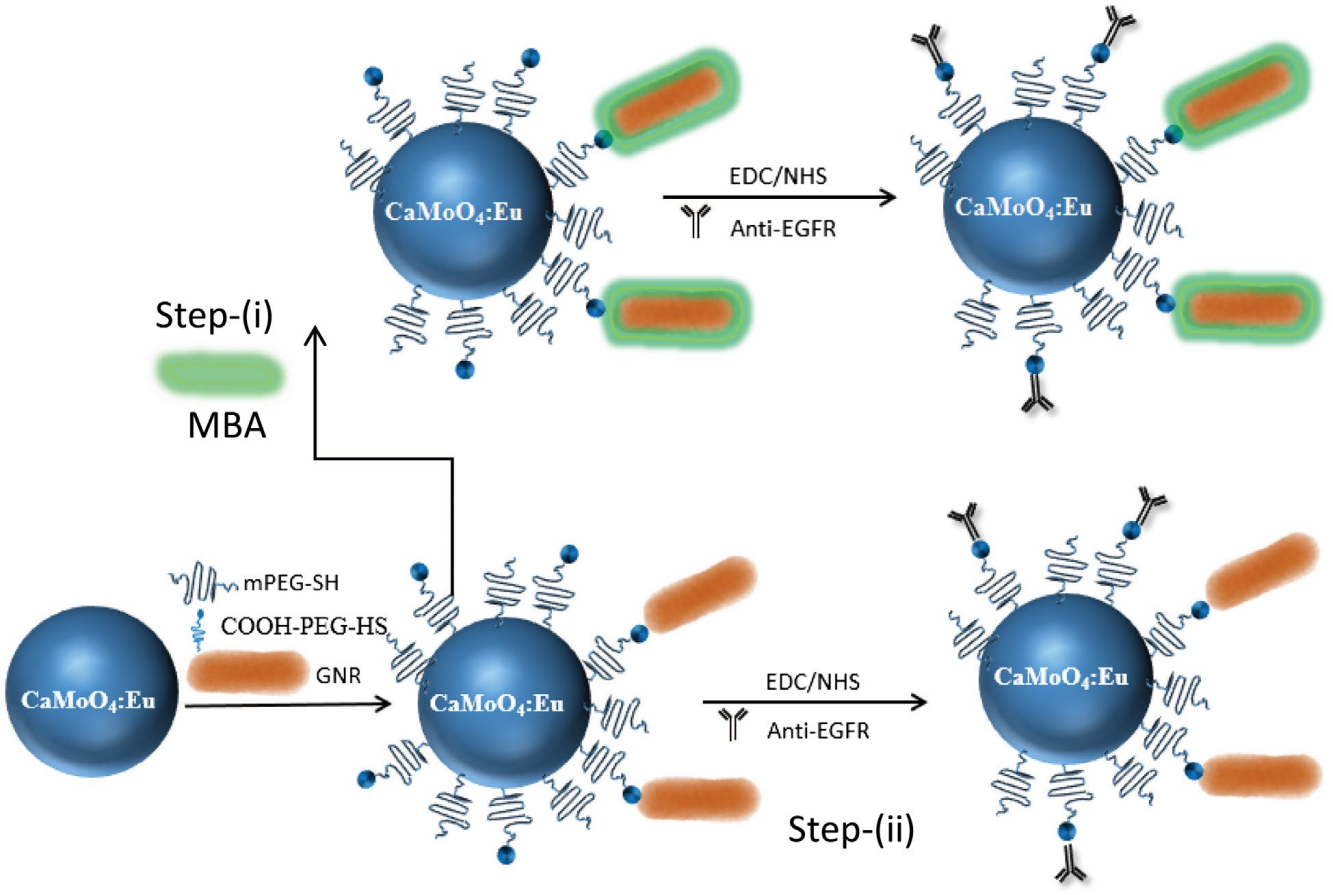
Schematic representation of surface modification and conjugation of Au nanorods on the surface of CaMoO_4_:Eu nanoparticles.

Herein, we demonstrate the potential use of CaMoO_4_:Eu@GNR (CMO:Eu@GNR) HNPs as multi-functional probes for optical imaging, SERS and PTT agent (Figure 1). The specificity of anti-EGFR Ab coated CMO:Eu@GNR are used for the enabled targeting of EGFR over-expressing of human lung cancer cells (A549 cell). Also, the effect of CMO:Eu@GNR on cellular biomechanics and biocompatibility of the cancer cells were studied. The SERS enhancement factor (EF), photothermal responses and efficiency of light-to-heat conversion of the CMO:Eu@GNR were evaluated. Also, we investigate the influence of HNPs on the PTT of A549, AML12, and white blood cells (WBC) cells illuminated at an 808 nm laser for *in vitro* cancer killing study.

## 2. Materials and methods

### 2.1. Materials

Calcium nitrate tetrahydrate, (Ca(NO_3_)_2_.4H_2_O, 99%, Alfa Aesar), ammonium molybdate (H_8_MoN_2_O_4_, 99.99%, Alfa Aesar, Ward Hill, MA, USA), europium(III) nitrate hydrate (Eu(NO_3_)_3_ .xH_2_O, 99.99%, Sigma Aldrich, St. Louis, MO, USA), oleic acid (Alfa Aesar), 1-octadecene (95%, Alfa Aesar), NaOH pellet (Merck, Kenilworth, NJ, USA), hydrochloric acid (HCl, Sigma-Aldrich), HS-PEG-COOH (MW = 5000, NANOCS, New York, NY, USA), mPEG-SH (MW = 5000, NANOCS), *N*-(3-dimethylaminopropyl)-*N*′-ethylcarbodiimide hydrochloride (C_8_H_17_N_3_HCl, MW = 191.7 g mol^–1^, Sigma-Aldrich) (EDC), *N*-hydroxysuccinimide (C_4_H_5_NO_3_, Sigma-Aldrich, MW = 115.09 g mol^–1^) (NHS), 4-mercaptobenzoic acid (MBA) (Sigma-Aldrich), anti-human epidermal growth factor receptor (EGFR) antibody (Thermo Fisher Scientific, Waltham, MA, USA), and phosphate buffered saline (1×) (PBS) (Thermo Fisher Scientific) were used for HNP synthesis. Human (*Homo sapiens*) lung carcinoma (A549 cells) (ATCC, USA), mouse hepatocyte cells (AML12, normal hepatocyte from liver tissue), ethylenediaminetetraacetic acid (EDTA) stabilized human whole blood was freshly obtained from Innovative Research (Novi, MI, USA), 0.5% trypsin-EDTA solution (Life Technologies), LIVE/DEAD viability/cytotoxicity assay kit (Life Technologies), Earle’s balanced salt solution (EBSS) (Life Technologies) and PBS were used for cell experiments.

### 2.2. Hybrid nanoparticle synthesis

CMO:Eu (2 at.%) NPs were synthesized by a thermolysis process: 0.0206 g of Eu(NO_3_)_3_ · xH_2_O, 0.1 g of NaOH, and 1.0 g of Ca(NO_3_)_2_.4H_2_O were dissolved in 2 ml distilled (DI) water. The solution was treated with 2 ml oleic acid (OA) and 18 ml 1-octadecene (ODE) and heated at 80°C for 1 h. In another beaker, 0.423 g of H_8_MoN_2_O_4_ was dissolved in 3 ml DI water, and 0.1 g of NaOH, 2 ml OA, and 18 ml ODE were added and the solution stirred at 80°C for 1 h. The two solutions were mixed under continuous stirring and heated at 80°C for 30 min, and then the reaction was refluxed at 309°C for 1 h. The resulting precipitate was collected by centrifugation at 6000 rpm after washing with ethanol.

Twenty mg of the CMO:Eu NPs was dispersed in 5 ml of 0.1 M HCl, and the mixture was sonicated for 1 h. To this, 2 ml of diethyl ether was added and sonicated for 30 min. The resulting solution was centrifuged at 6000 rpm for 15 min. The obtained precipitate was washed twice with ethanol and redispersed in 5 ml of PBS solution. To this, 20 mg of HS-PEG-COOH was added and sonicated for 1 h. The PEGylated capped NPs were collected by centrifugation and washed with PBS solution for three times to remove the excess of HS-PEG-COOH present in the sample. The final precipitate obtained was redispersed in a PBS solution. For the synthesis of HNPs, GNRs with 10 nm in diameter and 35 nm in length were purchased from Nanopartz, Loveland, CO, USA. First, 4 ml of the GNRs was centrifuged at 13,000 rpm for 30 min and then redispersed in PBS. Centrifugation was repeated for three times to reduce the excess of cetyl trimethylammonium bromide (CTAB) present on the surface of the GNRs. Four ml of GNRs dispersed in PBS was added to 1 ml of the PEGylated CMO:Eu NPs under continuous stirring and then sonicated for 1 h. The resulting solution was centrifuged, and the HNPs precipitated was collected. These particles were washed with a PBS solution for three times and redispersed in PBS.

### 2.3. Raman reporter labeling and antibody conjugation

The synthesized NPs were labeled with MBA by adding 200 μl of an MBA solution (2 mM) into 1 ml of the synthesized NPs solution. After 30 min sonication, the MBA-labeled CMO:Eu@GNR NPs were collected. For conjugating the anti-human EGFR antibody with the MBA-labeled CMO:Eu@GNR NPs, 10 μl HS-PEG-COOH of 1 mg ml^–1^ concentration was added into the MBA-labeled NPs. After 15 min sonication, 40 μl mPEG-SH of 1 mg ml^–1^ was added for 2 h incubation followed by 30 min sonication. The prepared NPs were centrifuged for 15 min at 13,000 rpm and then resuspended in water. Next, 10 μl EDC (10 mM) and 10 μl NHS (25 mM) were added and sonicated for 30 min. The prepared NPs were centrifuged for 15 min of 13,000 rpm and then resuspended in PBS. Then, the prepared NPs were labeled with antibody (20 μl, 0.2 mg ml^–1^) with 1 h sonication. After 15 min centrifugation at 13,000 rpm, the prepared NPs were resuspended in PBS and stored at 4°C for further experiments.

### 2.4. Characterization of synthesized NPs

Transmission electron microscopy (TEM) images and energy-dispersive X-ray spectroscopy spectrum (EDX) were collected using an FEI Titan 80–300 kV (S) TEM equipped with a spherical aberration (Cs) image corrector. All the images were collected at 300 kV. For the TEM measurements, the powder samples were ground and dispersed in methanol. A drop of the dispersed particles was placed over a carbon-coated copper grid and evaporated to dryness at room temperature.

UV–visible spectra were recorded using a Multiskan UV–visible spectrophotometer (Thermo Fisher Scientific). All the luminescence spectra were recorded using a Horiba FluoroMax-3 fluorescence spectrophotometer (HORIBA Scientific, Edison New Jersey, NJ, USA). A zeta potentiometer (ZetaPALS, Brookhaven Instrument, Holtsville, NY, USA) was used to measure the surface charge of the particles. Hydrodynamic diameter and particle size distributions of the HNPs were determined by dynamic light scattering (DLS) measurements using a DynaPro NanoStar (Wyatt Technology, Goleta, CA, USA) instrument at 25.0 ± 0.1°C. Disposable cuvettes were used for the measurements. 

The temperature changes of the CMO:Eu@GNR solutions irradiated by an 808 nm NIR laser (Xi’an Sampling Laser Technik Institute, Xi’an, China) were collected by a portable fiber optic thermometer (Qualitrol, Fairport, NY, USA). The photothermal images of the CMO:Eu@GNR solutions were recorded using an FLIR A20 camera (FLIR Systems, Inc., Wilsonville, OR, USA), and the laser power was measured using a handheld laser power meter (Edmund Optics, Barrington, NJ, USA).

### 2.5. Calculation of Raman enhancement factor of CMO:Eu@GNR-MBA

(1) $$EF=\;\frac{I_{SERS}}{I_{RS}}\;\times \frac{N_{RS}}{N_{SERS}}$$

Using the above equation, *N*_*SERS*_ was calculated from the results of TEM and concentration analyses. First, the laser-activated volume (*V*_*laser*_) in the micro-Raman experiment was calculated from the laser spot radius [*r*_*spot*_ = 0.61λ/NA = 0.53 μm; λ = 785 nm, NA (numerical aperture) = 0.9] and the penetration depth (*p*_d_ = *x* μm), resulting in a *V*_*laser*_ value of 0.89*x* μm^3^. From the commercial sample, the surface area and weight of one GNR (length = 38 nm, diameter = 10 nm) were determined as 1350 × 10^−6^ μm^2^, and 5.2 × 10^−14^ g, respectively. The concentration of the CMO:Eu@GNR-MBA solution was about 20 Au μg ml^–1^, which corresponds to 3.85 × 10^8^ GNRs ml^–1^ = 3.85 × 10^−4^ GNRs μm^–3^. Therefore, the CMO:Eu@GNR-MBA solution in *V*_*laser*_ contained 3.43 × 10^−4^ GNRs, which indicates that the SERS spectra were generated by 3.43 × 10^−4^ CMO:Eu@GNR-MBA molecules. On the other hand, the surface area of the MBA was 0.33 nm^2^ as a monolayer [25] and therefore the number of MBA molecules absorbed onto one GNR was approximately 4091 and consequently 1.4*x* MBA molecules, which is *N*_*SERS*_ in the below equation, were absorbed onto the GNRs present in *V*_*laser*_.

The density of MBA used in regular Raman detection was approximately 3.1 × 10^−6^ g ml^–1^. Thus, the number of MBA molecules in *V*_*laser*_ for regular Raman detection was 1.1 × 10^4^x MBA. Finally, we determined the Raman enhancement factor (EF) as:

$$R_{EF}=\frac{N_{RS}}{N_{SERS}}\times \frac{I_{SERS}}{N_{{}_{RS}}}=\frac{1.1\times 10^{4}}{1.4x}\times \frac{58,495}{925}\;\approx 5.0\times 10^{5}$$

### 2.6. Heat transfer efficiency of synthesized NPs

The change in the temperature of the HNPs was estimated by the heat input from the NIR laser via GNRs and heat dissipated into the ambient atmosphere, which can be expressed as follows:

(2) $$\sum_{i=2}m_{i}C_{i}\;\frac{\text{d}T}{\text{d}t}=Q_{in}-Q_{out}$$

where *m*_*i*_ and *C*_*i*_ are the mass and specific heat capacity of sample *i*, respectively. T is the temperature of the HNPs on NIR irradiation at time *t*. The mass of HNPs is significantly smaller than that of water (1 g), and the specific heat capacity of GNRs and water are 0.129 J g^−1^ K^−1^ and 4.18 J g^−1^ K^−1^, respectively.[26] By neglecting the specific heat capacity of GNR, Equation (2) can be modified as follows:

(3) $$C_{i}\;\frac{\text{d}T}{\text{d}t}=Q_{in}-Q_{out}$$

where *Q*_*in*_ = (*I*_*0*_ – *I*_*tr*_)*η* and *Q*_*out*_ = *∑hS[T(t*) – *T*_*o*_ *]*, *I* and *I*_*tr*_ are the NIR laser power before and after transmitting through the HNPs, *h* is the heat transfer efficiency, and S is the surface area of the interference between the HNPs and external environment. The increase in the temperature of the HNPs at any time t can be estimated as follows [26]:

(4) $$T\left(t\right)=T_{0}+\frac{(I_{0}-I_{tr})\eta }{mCB}\left(1-e^{-Bt}\right)$$

where *T*_*m*_ is the maximum stable temperature of the HNPs at which laser is turned off, *η* is the photothermal conversion efficiency, and *B* is the heat dissipation constant.

The dissipation constant (*B*) was calculated using the temperature decay profile after the laser was turned off as follows:

(5) $$T\left(t\right)=T_{0}+\left(T_{m}-T_{0}\right)e^{-Bt}$$

In thermal equilibrium condition, Q_in_=Q_out_ i.e. $\eta =mCB\frac{\Delta T}{\Delta I}whereallsymbolshavetheirusualmeanings.$

### 2.7. Cell culture and NP treatment

A549 cells (ATCC, Manassas, VA, USA) were cultured in F-12 k medium containing 10% fetal bovine serum at 37°C with 5% CO_2_ in a humidified atmosphere. Mouse hepatocyte cells (AML12, normal hepatocyte from liver tissue) purchased from American Type Culture Collection (ATCC) were cultured in a 1:1 mixture of Dulbecco’s modified Eagle’s medium and Ham’s F12 medium (ATCC) with 0.005 mg ml^–1^ insulin, 0.005 mg ml^–1^ transferrin, 5 ng ml^–1^ selenium, 40 ng ml^–1^ dexamethasone (Sigma-Aldrich, St. Louis, MO, USA) and 10% fetal bovine serum (ATCC, Manassas, Virginia, USA) at 37°C with 5% CO_2_ in a humidified atmosphere.

Both cells were passaged at 70–90% confluency using 0.5% Trypsin-EDTA solution, and the cell number was estimated by a hemocytometer to be 1 × 10^5^ cells ml^–1^. A549 and AML12 cells (1 × 10^5^ cells ml^–1^) were treated with 100 μl prepared NPs (20 μg ml^–1^) for 2 h incubation at 37°C. Then, cells were washed to remove non-bound NPs. The binding of the NPs onto the cells was verified by fluorescence and SERS spectra. The fluorescence images were captured under a fluorescence microscope with a DP30BW CCD camera (Olympus IX71, Olympus America Inc., Center Valley, PA, USA) with an excitation at 450 nm and an emission at 630 nm. EDTA stabilized human whole blood were freshly obtained from Innovative Research. Whole blood and serum were used for white blood cell (WBC) count analysis.

### 2.8. Atomic force microscopy

A549 cells were detected by atomic force microscopy (AFM) in the contact mode (PicoPlus, Agilent Technologies, Santa Clara, CA, USA) controlled by Picoview software, Agilent Technologies, Santa Clara, CA, USA. The AFM probe was made of silicon nitride and had a 20 nm tip radius (Bruker Corp., Billerica, MA, USA); its spring constant was calibrated as 0.06–0.10 N m^–1^, and the deflection sensitivity was 30–40 nm V^–1^. The biomechanical properties (Young’s modulus and adhesion force) of cells were calculated using Scanning Probe Image Processor (SPIP) software (Image Metrology, Hørsholm, Denmark) by Sneddon’s modification of the Hertz model from the force curves for the elastic indentation in a flat and soft sample.[27,28] The model describes the relationship between the applied loading force *F* and the indentation depth *δ*:

$$F=\frac{2}{\pi }\times \tan\;\left(\alpha \right)\times \frac{E_{cell}}{1-\gamma^{2}}\times \delta^{2}$$

where *E*_*cell*_: Young’s modulus; F: loading force; *γ*: Poisson ratio (its value was 0.5); and *α*: tip half cone opening angle (its value was set to 36°). The force was obtained by the cantilever deflection *d*(*z*) and the spring constant of the cantilever *k*: *F* = *k* × *d*(*z*). The indentation depth was calculated from the *z*-height and the cantilever deflection: *δ* = *z* – *d*(*z*). The Young’s modulus were obtained from the force curves transformation and the linear regression fitted by the Hertz model.[29] For each group, at least 25 force curves of each cell (the total cells are over 20) were detected, and the detection was accomplished within 2 h to approximate cellular physiological conditions. For deflection and 3D view images, the AFM images were imported into a WSXM software (Nanotec, Madrid, Spain).

### 2.9. SERS measurements of cells treated with synthesized NPs

SERS spectra were recorded using a Renishaw inVia Raman spectrometer (WIRE 3.3 software, Renishaw, Wotton-under-Edge, UK) equipped with a 300 mW, 785 nm NIR laser. Cells were cultured on magnesium fluoride (MgF_2_, United Crystals Co., Port Washington, NY, USA) and imaged in EBSS through a 63 × (NA = 0.90) water immersion objective (Leica Microsystems, Buffalo Grove, IL, USA). For Raman streamline mapping, the data were acquired at one accumulation with 10 s exposure, and the peak at 1078 cm^−1^ from MBA was selected for mapping. On each group, the cells were detected within 2 h at room temperature. Renishaw Wire 3.3 software (Renishaw) performed for Raman spectra baseline corrected, spectral smoothed, and normalized at maximum peaks. The processed spectra were imported to OriginPro 9 software (OriginLab Co., Northampton, MA, USA) for analysis.

### 2.10. NIR photothermal therapy on cells

For NIR PTT, A549 and AML12 cells (∼1 × 10^5^ cells ml^–1^) were incubated with about 100 μl prepared NPs (20 μg ml^–1^ CMO:Eu@GNR-MBA-Ab and CMO:Eu@GNR-MBA, respectively) for 2 h incubation at 37°C. Next, the cells were rinsed with PBS thrice and then exposed to the 808 nm laser irradiation at 1 W cm^–2^ power densities for 5 min. For the cell viability test, the cells with triplicates were stained using a LIVE/DEAD viability/cytotoxicity assay kit according to the instructions. After staining, the cells were imaged using a fluorescence microscope equipped with a DP30BW CCD camera (Olympus IX71) at 10 × objective to analyze the relative proportion of live/dead cells.

### 2.11. Statistics analysis

Data are presented as mean ± standard deviation of error. Differences were considered significant at *p*<0.05. OriginPro 9 software was used for one-way ANOVA for significance test.

## 3. Results and discussion

### 3.1. Characterization of HNPs

**Figure 2.**
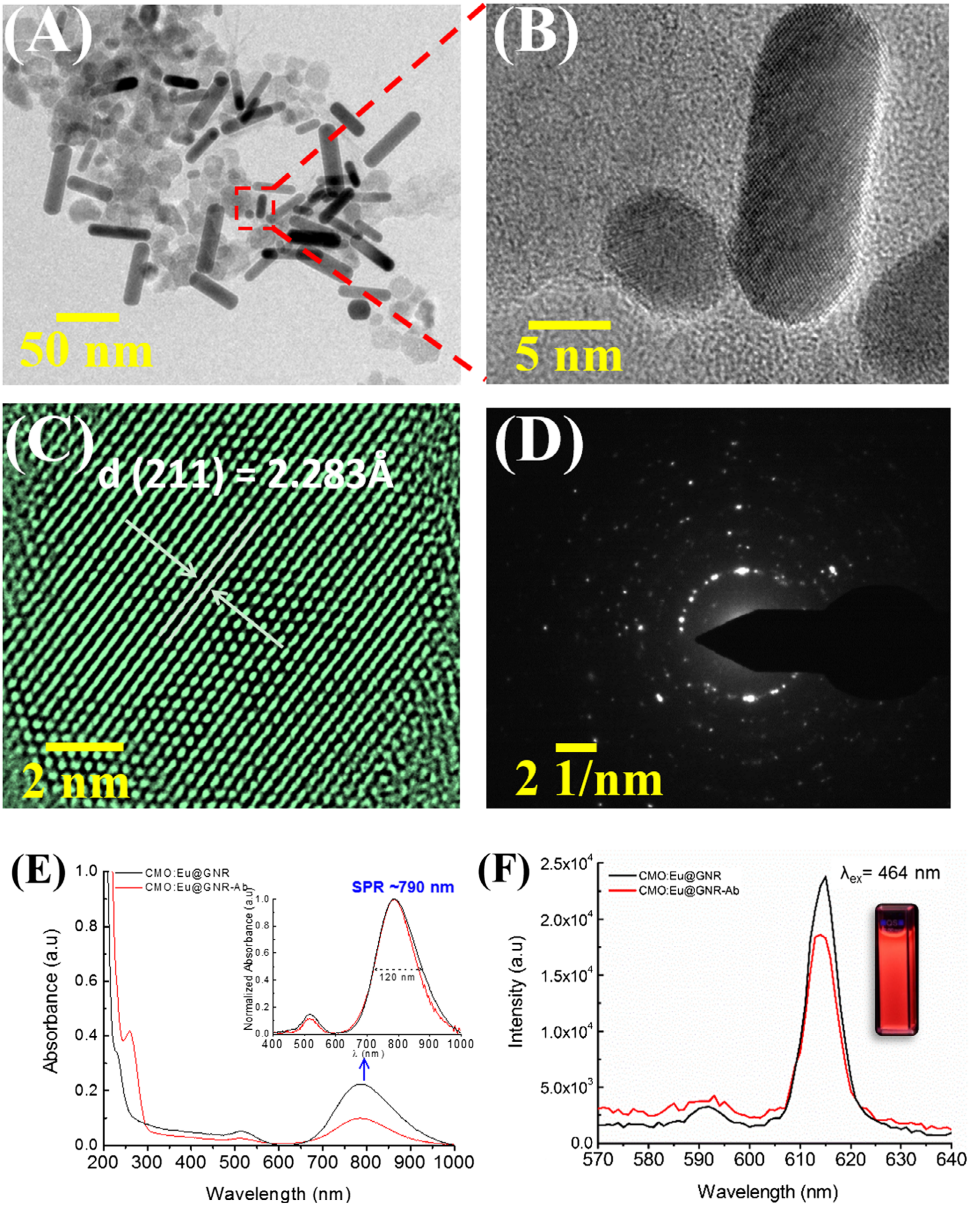
(A) TEM and (B) HRTEM images of CMO:Eu@GNR. (C) HRTEM image of CaMoO_4_:Eu NPs and (D) SAED pattern for (A). (E) UV–visible and (F) photoluminescence (λ_ex_ = 464 nm) spectra of CMO:Eu@GNR without (black) and with Ab (red). Inset in (E) shows the comparison of normalized absorption spectrum at an SPR of 790 nm. Digital photograph of the CaMoO_4_:Eu NPs dispersed in PBS under a UV-lamp (λ_ex_ = 254 nm), shown in the inset of (F).

TEM and high-resolution TEM (HRTEM) images of the CMO:Eu@GNR NPs and selected area electron diffraction pattern (SAED) are shown in Figure 2(A)–(D). The TEM image confirms the formation of hybrid nanostructures where GNRs are attached to the surface of CaMoO_4_:Eu NPs (Figure 2(B)). The average sizes of CaMoO_4_:Eu were found to be 10–15 nm, and GNRs had an average diameter of 8–12 nm and a length of 40 nm. The distance between lattice planes 2.283 Å corresponding to the lattice spacing in the (211) plane of tetragonal CaMoO_4_, JCPDS card No. 29–0351 (National Institutes of Health, Bethesda, MD, USA; Figure 2(C)) was calculated using ImageJ Software v1.47 (Figure S1). The bright circular spots in the SAED patterns confirmed the presence of both CaMoO_4_ and GNRs phases in the CMO:Eu@GNR (Figure 2(D)). Furthermore, the energy dispersive X-ray analysis (EDX) spectrum (Figure S2) confirms the presence of Ca, Mo, O, Eu, and Au elements in the hybrid sample. Figure 2(E) shows the UV–visible spectra of CMO:Eu@GNR with and without Ab between 200–1000 nm. Three characteristic peaks were observed at 260, 530, and 790 nm. The absorption band 260 nm was assigned to the Mo–O charge-transfer band (CTB),[30] and the bands 530 and 790 nm can be attributed to the surface plasmon resonance (SPR) of GNRs.[3] The inset of Figure 2(E) shows a comparison of the normalized SPR absorption spectrum between 400 and 1000 nm. Figure 2(F) depicts the photoluminescence spectra of the CMO:Eu@GNR with and without Ab coating at a fixed excitation of 464 nm (^7^F_0_→^5^D_2_) and shows a strong red luminescence 612 nm. The inset shows a digital photograph of HNPs under UV light. The excitation spectrum (λ_em_ = 615 nm) and emission spectra at different excitations of HNPs are shown in Figure S3. HNPs show a strong excitation spectrum at 275 nm, which is assigned to O→Mo CTB (Mo–O CTB), and two sharp peaks at 394 and 464 nm are assigned to the ^7^F_0_→^5^L_6_ and ^7^F_0_→^5^D_2_ transitions of Eu^3+^, respectively.[8]

Furthermore, hybrid nanomaterials with fluorescence in the red region and NIR-SPR properties have become increasingly attractive in the theranostic of cancer, combining both diagnostic and therapeutic functions. Due to deep tissue penetration of NIR radiation, it can be used as an emerging tool in the fight against cancer.[31] Antibody conjugation to HNPs was further confirmed by red shift in Mo–O CTB (15 nm) and SPR band (0.5 nm) (Figure 2(E)). The decrease in SPR band absorption for the Ab-conjugated NPs implies that the surface of the GNRs has a different environment than Ab-free NPs. EI-Sayed et al. [32] performed a detailed analysis with and without anti-EGFR conjugated Au NPs to distinguish between cancerous and noncancerous cells using red shift^32^. Moreover, Ab conjugated Au NPs were specifically and homogeneously bound to the surface of the cancer cells with 600% greater affinity than to the noncancerous cells.[32] Furthermore, a slight decrease in the luminescence intensity of Eu^3+^ ion was observed after Ab conjugation (Figure 2(F)). Asymmetric ratio (A_21_ = ∫^5^D_0_→^7^F_2_/∫^5^D_0_→^7^F_1_) values without and with Ab coated NPs are found to be 8.6 and 5.3, respectively. The colloidal stability of the NPs was estimated using a zeta potential (the potential close to the particle surface and thus the electrostatic stabilization) in PBS solvent. The average zeta potential of the GNR particles in a CTAB solution was found to be 41.12 mV. The value slightly decreased when the particles were dispersed in PBS (35.5 mV), whereas the PEGylated CMO:Eu NPs showed a negative zeta potential of 29.5 mV. The CMO:Eu@GNR HNPs showed a zeta potential of 27.6 mV. This indicates that positive charge is present on the surface of HNPs. It confirms the high stability of the particles in PBS. Figure S4 shows the comparison of zeta potential values at pH 7. Further, to confirm the stability of CMO:Eu@GNR in PBS, luminescence of the NPs was measured in every 24 h for 10 days. There is a slight decrease in the luminescence stability signal (5%) after 10 days. Moreover, the hydrodynamic size distribution of the HNPs was confirmed by dynamic light scattering experiments (DLS) (Figure S5). These results indicate the use of these HNPs for potential bioapplications.

The biocompatibility of the HNPs was investigated using the LIVE/DEAD viability/cytotoxicity assay kit (Figure S6). Cells have high viability (>90%) at lower concentrations of the CMO:Eu@GNR (2.5–20 μg ml^–1^) incubated for 24 h and decreased to 84% as the concentration increased to 40 μg  ml^–1^. The decrease in cell viability at high concentration of CMO:Eu@GNR can be attributed to the production of hydroxyl radicals from luminescent functionalized CaMoO_4_:Eu.[33] Hydroxyl radicals can generate reactive oxygen species (ROS), causing cellular apoptosis.[34,35]

We synthesized CaMoO_4_:Eu NPs having a strong luminescence 615 nm and conjugated with GNRs having NIR absorption 790 nm. When these HNPs are uptaken at the tumor site, the temperature increase (42°C) on NIR irradiation can lead to the cancer cells being killed. HNPs synthesized in this way have a great advantage in PTT tumor ablation. The HNPs were coated with anti-EGFR Ab for the selective targeting of A549 cancer cells. Indeed, the HNPs are good candidates for the development of PTT and imaging agents due to its easy access, simple conjugation procedures and low toxicity.

### 3.2. Photothermal properties of HNPs

**Figure 3.**
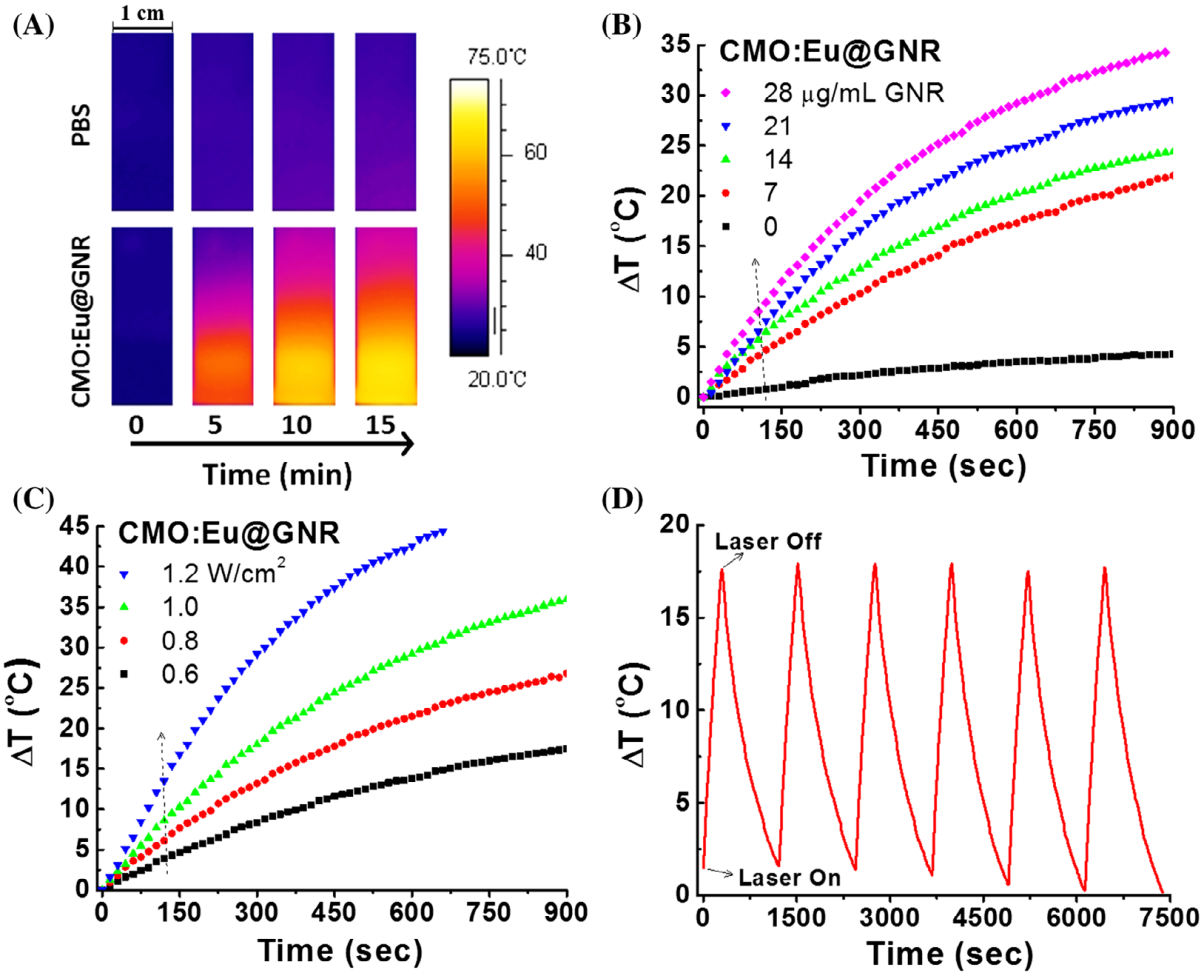
(A) Infrared images of PBS and CMO:Eu@GNR aqueous solutions exposed to 808 nm laser (1 W cm^−2^) for 900 s recorded at different time intervals. (B) Photothermal responses of CMO:Eu@GNR NPs at different concentrations in aqueous solution for 900 s NIR laser (808 nm, 1 W cm^–2^). (C) Photothermal responses of 808 nm laser irradiation with different power densities for 900 s at fixed CMO:Eu@GNR NP concentration (28 μg ml^–1^ GNR). (D) Temperature change of CMO:Eu@GNR solution with 28 μg ml^–1^ GNR at 1 W cm^–2^ 808 nm laser irradiation over six LASER ON/OFF cycles. Experiment was carried out at room temperature.

The PTT abilities of HNPs were investigated using 808 nm NIR laser irradiation. Figure 3(A) shows a thermal image of PBS and CMO:Eu@GNR solution placed in a 1 cm quartz cuvette using a forward looking infrared (FLIR) thermal imaging camera on irradiation with a NIR laser (1 mm spot size, 1 W cm^–2^) after 900 s at room temperature. As the irradiation time increased, the color of the thermal images of HNPs is gradually changed from blue to bright yellow (high temperature). In contrast, the thermal images for the PBS solution changed slightly over time as compared to those HNPs. Thermal images confirm that the NIR light could be absorbed by the CMO:Eu@GNR and converted to heat energy. Figure S7 shows a digital photograph of the photothermal setup used for the measurement of PTT in this study. It consists of a fiber-optic thermocouple temperature sensor (accuracy ± 0.1°C) for temperature measurement, a FLIR thermal imaging camera, and an 808 nm NIR laser.

Figure 3(B) and (C) show the temperature kinetic curves at different concentrations (excitation 1.0 W cm^–2^) and excitation powers (28 μg ml^–1^ of GNRs) of the CMO:Eu@GNR irradiated with NIR laser for 900 s (Δ*T* is the temperature change, sample temperature 27°C). The temperature of the HNPs solution exponentially increases with the concentration of GNRs, and the similar temperature increasing profile is observed with the increase of excitation power. CMO:Eu@GNR achieved a PTT temperature of 42°C (Δ*T* = 15°C) in 210, 258, 377, and 481 s for 28, 21, 14, and 7 μg ml^–1^ of GNRs, respectively (Figure 3(B)). In the case of 0 μg ml^–1^ of GNRs (pure PBS), Δ*T* was found to be 4.4°C in 900 s, which is 87.5% lesser than PTT temperature obtained using CMO:Eu@GNR (28 μg ml^–1^ GNRs). The heating ability of the CMO:Eu@GNR at various laser irradiation powers for 28 μg ml^–1^ of GNRs concentration is shown in Figure 3(C). The required PTT temperature of 42°C (Δ*T* = 15°C) was obtained in 136, 237, 351, and 673 s for 1.2, 1.0, 0.8, and 0.6 W cm^–2^ of NIR laser power, respectively. In the case of 1.5 W cm^–2^, PTT temperature was acquired in 95 s (Figure S8(A)). Thus, by increasing the NIR laser power, the time required for the desired PTT temperature can be decreased. Moreover, the increase in the temperature of the PBS solution was significantly less than required PTT temperature.

Heat conversion efficiency (*η*) can be determined by plotting Δ*T* versus Δ*I* as reported by Pinchuk et al. [26,eqs (2–6)]. The value of heat dissipation rate constant (*B*) was further analyzed using the cooling temperature profile when the laser was turned off (Figure S8(B)). The natural log of (T(t)–T_0_)/(T_m_–T_0_) as a function of time after the laser was turned off is shown in Figure S8(C)). The average value of *B* was found to be 1.58 × 10^−3^ s^−1^ by linear fitting to Figure S8(C) with *R* ^2^ = 0.99923. Pinchuk et al. [26] also reported a *B* value of 4.66× 10^−3^ s^−1^ for spherical Au particles with an SPR of ~530 nm. It was reported that the value of *B* depended on the volume of the NPs in the cuvette and was almost independent of the amount of the NPs present in the sample^26^. Figure S9 shows the linear relationship between Δ*T* and Δ*I*. The *η* value was calculated from the slope of Figure S9 as 25.6%. However, a slight deviation in the Δ*T* was observed at a higher NIR laser irradiation power.

To understand the photostability of HNPs, six cycles of ON/OFF NIR laser irradiations were performed (1 W cm^–2^ for 300 s (laser ON), followed by naturally cooling for 900 s (laser OFF) (Figure 3(D)). It was found that the temperature (Δ*T*) increased by 17.6°C in the first laser ON condition of the CMO:Eu@GNR (GNR concentration 28 μg ml^–1^). During six cycles of laser ON/OFF, the temperature elevations remained almost the same as in the first cycle within the limits of error bar, indicating the good photostability of HNPs. Furthermore, the effect of laser ON/OFF on luminescence emission was measured (λ_ex_ = 464 nm) on the start and end of each cycle. A slight decrease in the emission of <2% was observed at the end of the sixth cycle.

HNPs with plasmon-enhanced fluorescence properties have attracted much attention as imaging nanoprobes for PTT due to their small size and deeper tumor permeation. Our HNPs have PTT conversion efficiency of 25.6% and a sharp luminescence peak at 615 nm. Recently, Sun et al. [36] synthesized GNRs and gold nanostars (GNSs) with strong NIR absorption of ~800 nm^36^. They concluded that pure GNRs show a higher *η* value, which varies in the range 69.7–94.2%. It is well known that pure Au nanorods and nanostars exhibit strong PTT activity (100%).[37] Also, GNRs shows high absorption cross section as compared to carbon nanotubes, quantum dots and organic dyes.[38] Self-assembled WO_3−x_ hierarchical nanostructures ranging from 700 to 1400 nm were prepared with *η* 28% by Hu et al. [39], and the same research group prepared CuS NPs with *η* 38%.[16] Although various nanostructures [16,36,39] have been evaluated as PTT agents, HNPs offer additional favorable properties that enable their use for cancer therapy. More importantly, the HNPs not only have a high *η* value, but also have good NIR photostability (Figure 3(D)) and fluorescence properties (Figure 2(F)). These results demonstrate that the CMO:Eu@GNR could be used as a photothermal and imaging agent for cancer therapy applications.

### 3.3. Cell biomechanical properties

**Figure 4.**
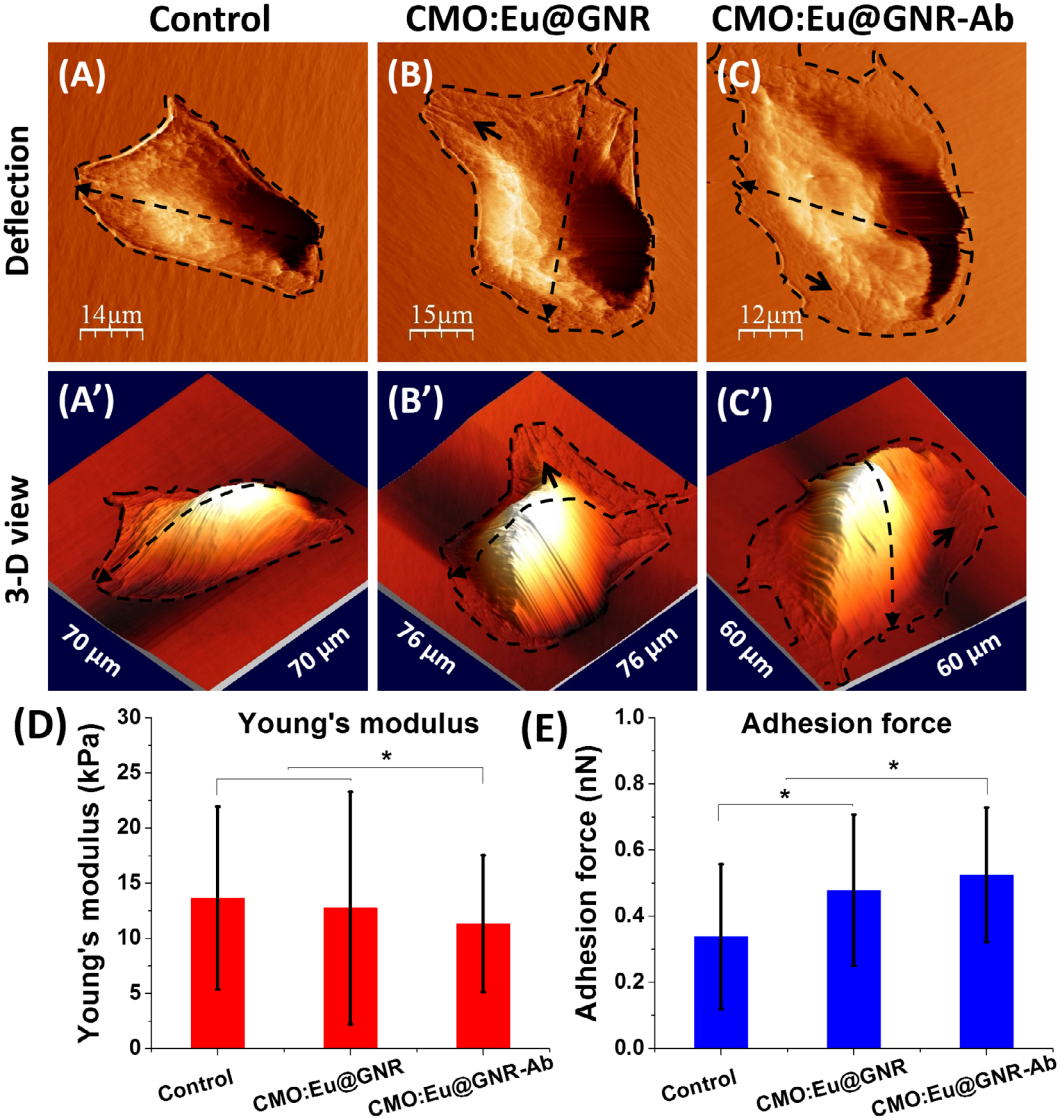
Atomic force microscopy detection of A549 cells (A) without treatment, (B) treated with CMO:Eu@GNR, or (C) CMO:Eu@GNR-Ab for 2 h: (A′–C′) are 3D view images of (A–C); (D) Young’s modulus and (E) adhesion force of cells. Error bar: standard deviation of the mean, * means *p* < 0.05.

To investigate the interactions between cells and the HNPs (without and with Ab), the cellular morphological effects were observed by AFM and compared with control cells (Figure 4). In case of the control group (Figure 4(A) and (A′)), its surrounding cytoskeleton structures were less visible than the NPs treated groups (Figure 4(B), (B′), (C) and (C′)). The short black arrows in these treated groups indicate the filamentous actin bundles, which suggest that the mechanical properties of cells vary with the interaction of HNPs.

Figure S10 shows the histograms of the Young’s modulus and adhesion force from control live A549 cells and the cells treated with CMO:Eu@GNR and CMO:Eu@GNR-Ab NPs using over 500 force–distance curves for each group to estimate the Young’s modulus and adhesion force. A comparison of biomechanical properties of different groups is shown in Figure 4(D) and 4(E). The control group has the largest Young’s modulus, 14 ± 8 kPa, while the CMO:Eu@GNR-Ab group has the lowest Young’s modulus, 11 ± 6 kPa. Furthermore, the CMO:Eu@GNR-Ab group has the largest adhesion force, 0.5 ± 0.2 nN, and the control group has the lowest adhesion force, 0.3 ± 0.2 nN. These results implied that the CMO:Eu@GNR-Ab has significant effects on the cellular biomechanics. One-way ANOVA for significance test was applied (*means *p* < 0.05; data were presented as mean ± standard deviation of error).

Biomechanical properties played important roles in cellular morphogenesis, focal adhesion, motility, and metastasis,[40–42] but also useful in medicine to understand the formation and stage of tumor development. The biomechanical properties of the cancer cells were investigated at single living cell level on incubation with HNPs. The biomechanical values of the control group were similar to our previous studies.[43,44] Our AFM results (Figure 4) revealed that the interaction of HNPs with cells showed more surrounding cytoskeleton structures, much softer cell membrane and increased surface adhesion force compared to control cells. These alterations in cell topography and biomechanics indicate that the HNPs affected the cellular biophysical properties within a short time (2 h) under similar experimental conditions.

### 3.4. Fluorescence imaging of HNPs treated cells

**Figure 5.**
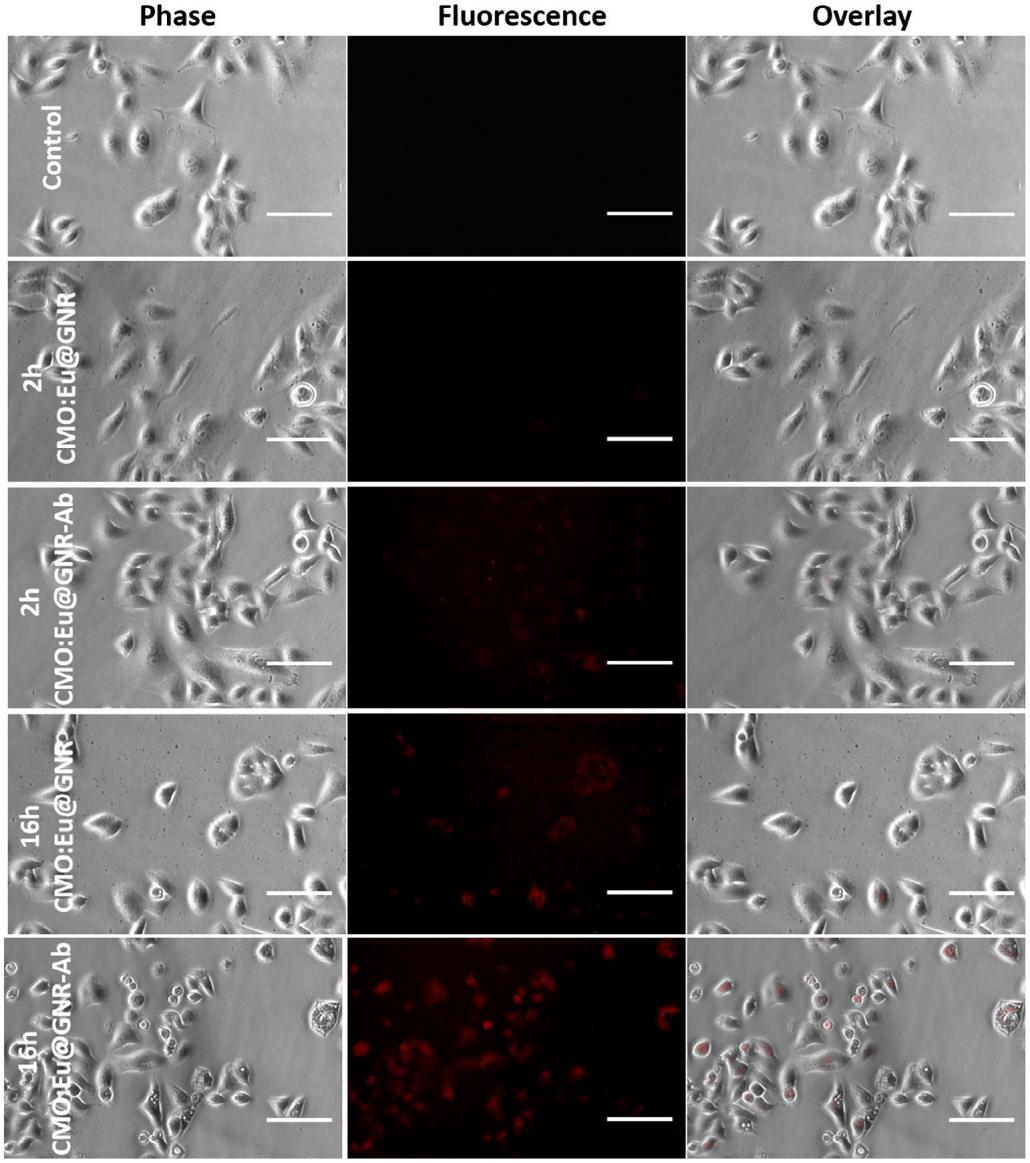
Phase, fluorescence, and overlay images of A549 cells without treatment and treated with CMO:Eu@GNR or CMO:Eu@GNR-Ab NPs for 2 h and 16 h. Scale bar: 100 μm.

To investigate the bioimaging application, CMO:Eu@GNR and CMO:Eu@GNR-Ab were incubated with A549 cells for 2 and 16 h, respectively. Red fluorescence from the CMO:Eu@GNR (λ_em_ = 615 nm) was observed from A549 cells on excitation 464 nm (^5^D_2_ level of Eu^3+^ ion). Figure 5 shows phase contrast, fluorescence, and overlap of phase contrast and fluorescence images of control, A549 cells incubated with CMO:Eu@GNR NPs for 2 and 16 h (with and without Ab). It was found that the fluorescence intensity from the cells after 16 h incubation >2 h incubation; and no fluorescence was observed from the control cells under similar conditions. The increase in the fluorescence intensity with time may be due to the more uptake of the CMO:Eu@GNR by the A549 cells. Ansari and co-workers [45] recently demonstrated the bioimaging applications of SiO_2_@Eu(OH)_3_ core-shell microspheres with a size of 392 nm for 24 and 48 h incubation time. The Ab-conjugated CMO:Eu@GNR after 16 h incubation showed the strongest fluorescence compared to other groups. This result showed the specificity of CMO:Eu@GNR-Ab as compared to the control and CMO:Eu@GNR groups. The fluorescence emission from Eu^3+^ doped NPs was similar to the previous studies that used Ab-coated NPs for enhanced imaging.[45–47]

### 3.5. SERS measurement

Cells on MgF_2_ were stained with Calcein AM (green, live cells)/ethidium homodimer-1 (red, dead cells) after 785 nm laser exposure in Raman measurement (within 2 h). Few dead cells were found as shown in Figure S11, suggesting a negligible photodamage effect from NIR laser in Raman instrument, which was similar to the previous report.[48]

**Figure 6.**
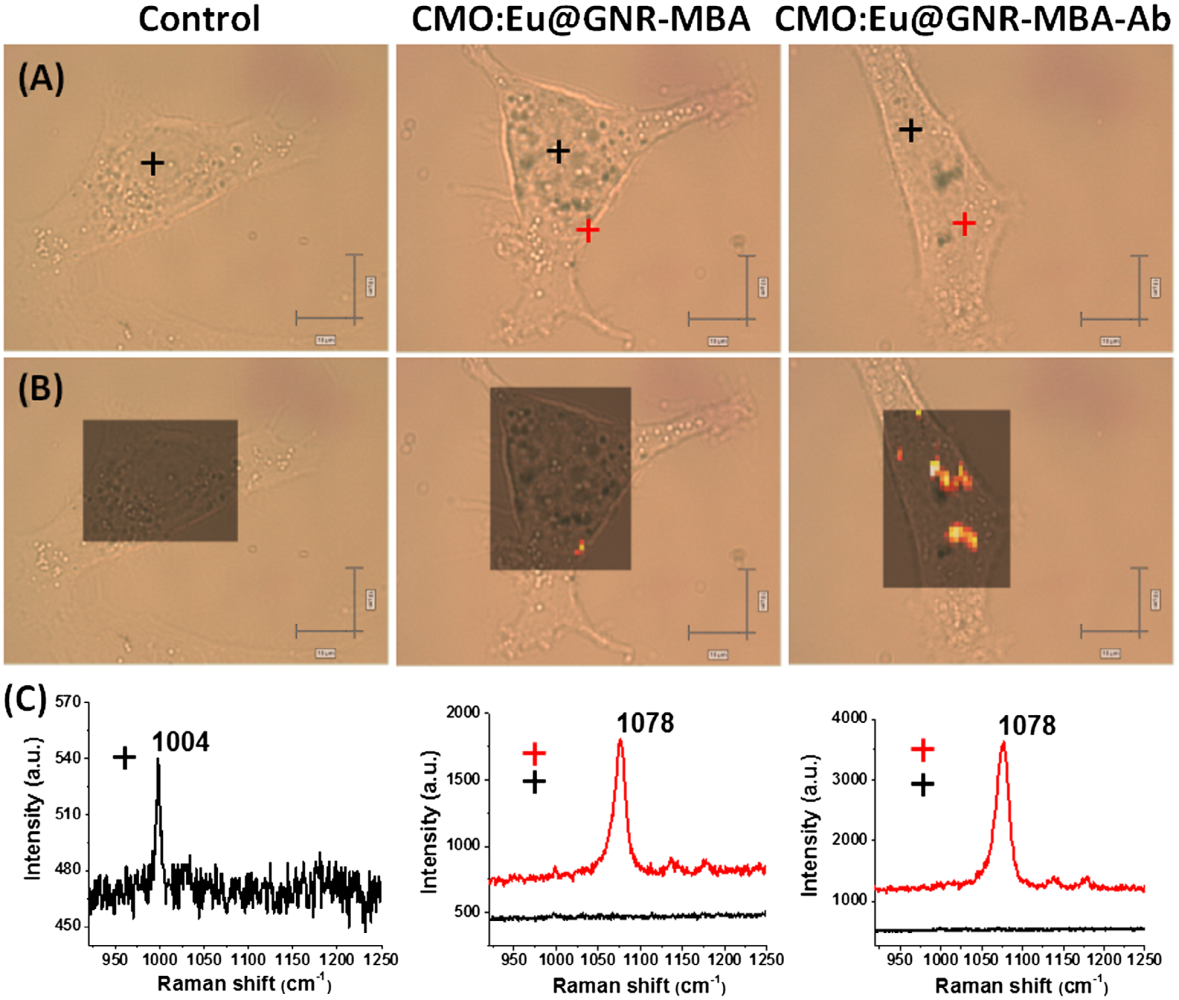
(A) Raman bright-field images of A549 cells without treatment and treated with CMO:Eu@GNR-MBA or CMO:Eu@GNR-MBA-Ab for 2 h (peak at 1078 cm^−1^ from MBA was selected for mapping). Scale bar: 10 μm (horizontal), 5 μm (vertical). (B) Raman streamline mapping and (C) the corresponding Raman spectra of A549 cells without treatment and treated with CMO:Eu@GNR-MBA or CMO:Eu@GNR-MBA-Ab NPs for 2 h.

The HNPs were conjugated with Ab for the enhancement of specificity. Figure S12 shows the normalized Raman spectra of CMO:Eu@GNR-MBA NPs with (black) and without Ab (blue) revealing no significance difference between them. Figure 6 shows the Raman bright-field image, Raman streamline mapping and Raman spectrum (900–1250 cm^−1^) from SERS negative (black cross) and SERS positive (red cross) of single live A549 cell incubated with CMO:Eu@GNR. Raman mapping for a live cell was performed by selection of 1078 cm^−1^ (a characteristic peak from Raman reporter molecule MBA). The Raman spectra obtained from SERS positive and negative spots are shown in Figure 6(C). The SERS positive spectra from both CMO:Eu@GNR-MBA and CMO:Eu@GNR-MBA-Ab groups show a strong characteristic peak from MBA at 1078 cm^−1^. The order of strong Raman mapping pixel intensity is found to be control < CMO:Eu@GNR-MBA < CMO:Eu@GNR-MBA-Ab. The bright color spots in the Raman mapping indicates the distribution of EGFR biomarkers on single live cell (Figure 6(B)). The high pixel intensity in Raman mapping for the CMO:Eu@GNR-MBA-Ab group confirms the higher cellular distribution of HNPs compared to non-Ab group. A few CMO:Eu@GNR are still distributed around the cellular membrane edges in the CMO:Eu@GNR-MBA group due to nonspecific binding. In contrast, more NPs were bound to the cells in the CMO:Eu@GNR-MBA-Ab group. However, no SERS signal was detected from the control group (no NPs treatment) except a lowest intensity characteristic peak 1004 cm^−1^, which is assigned to the phenylalanine from the cell.[49] CMO:Eu@GNR were also applied to the AML12 for SERS detection, as shown in Figure S13. It was found that a few NPs were distributed around AML12 live cell membrane of both CMO:Eu@GNR-MBA and CMO:Eu@GNR-MBA-Ab groups, indicating nonspecific binding of NPs. The spectra from Figure S13(C) confirmed these Raman positive spots were CMO:Eu@GNR due to presence of the characteristic peak from Raman reporter MBA molecule. These SERS results compared the spectral differences among the three groups, illustrating the specificity of the Ab-conjugated NPs and the distribution of these NPs.

HNPs-MBA shows high sensitive SERS properties, which arise from the interaction of MBA molecules with GNRs. Several factors (e.g. substrate types, aspect ratios, plasmon absorption, reporter molecules, and excitation source) [50] may affect the value of EF. The average EF value (5.0× 10^5^) of HNPs was similar to previous reports.[50,51] Raman streamline mapping in Figures 6 and S13 consisting of over 1000 spectra detected over 80% of the cell area. HNPs without Ab are partly attached to the cellular surface, suggesting that there was still nonspecific binding to cells due to long incubation time. Moreover, the nonspecific cellular binding of CMO:Eu@GNR NPs was significantly less than that of CMO:Eu@GNR-Ab with high specificity.

### 3.6. Photothermal treatment of cells

**Figure 7.**
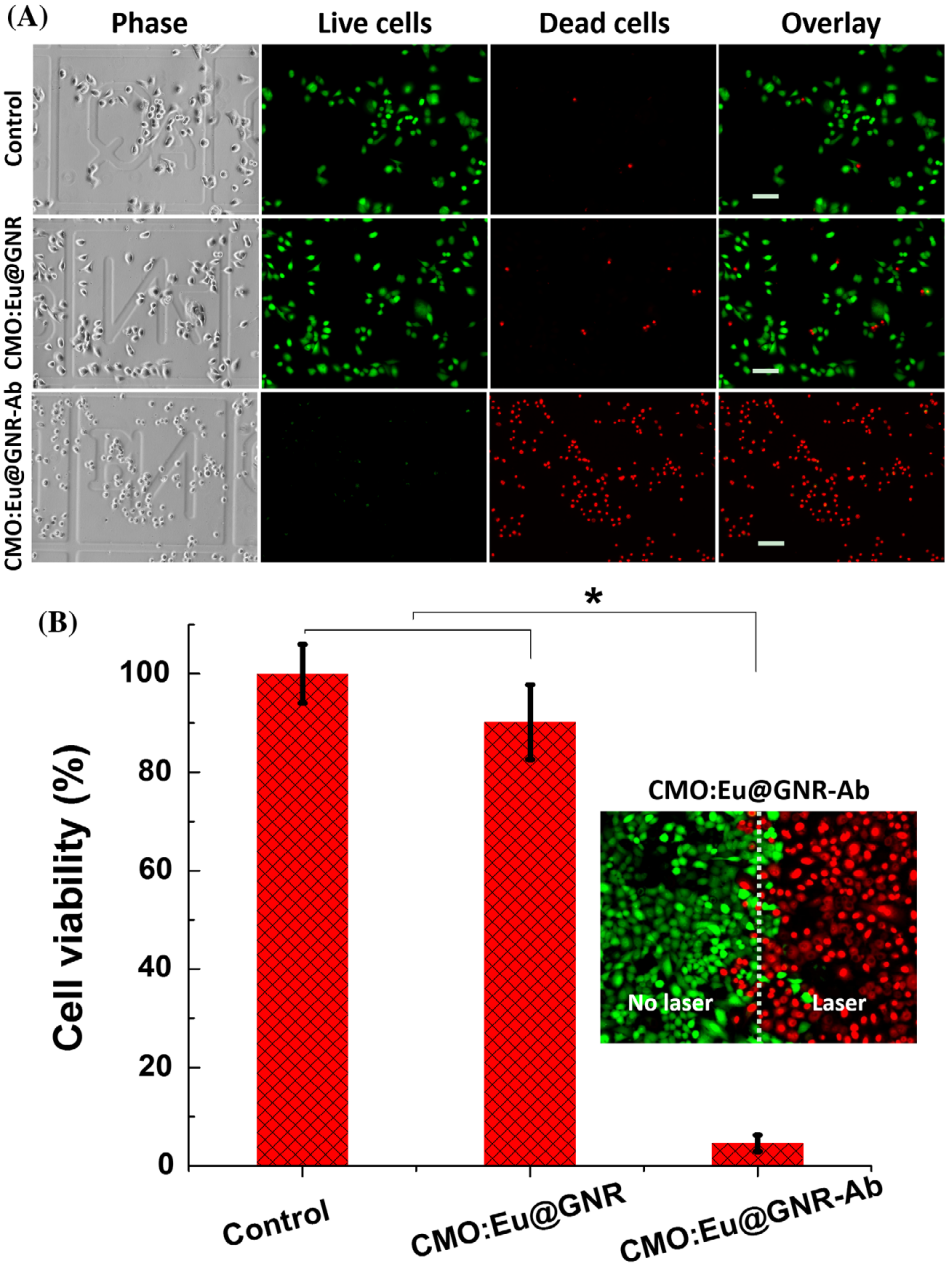
(A) Photothermal therapy. A549 cells were incubated without NPs (control), with CMO:Eu@GNR or CMO:Eu@GNR-Ab for 2 h; after that, cells were irradiated under 1 W cm^–2^ 808 nm laser for 5 min (green: live cells; red: dead cells. Scale bar: 100 μm). (B) Cell viability of A549 cells without treatment and treated with CMO:Eu@GNR or CMO:Eu@GNR-Ab for 2 h; after that, irradiation for 5 min under 1 W cm^–2^ 808 nm laser. Inset shows the fluorescence image of A549 cells treated with CMO:Eu@GNR-Ab NPs for 2 h, then irradiated without/with laser; error bar: standard deviation of the mean, * means *p* < 0.05.

NPs with strong NIR absorption are considered to be a relatively noninvasive and effective treatment of cancer compared to the current cancer treatments (chemotherapy, radiotherapy, surgery, and so on), which usually result in severe adverse effects and cancer recurrence.[52–55] In PTT, the malignant cells were killed by localized hyperthermia generated by the conversion of absorbed light to heat. The distribution of cancer biomarker EGFR (via CMO:Eu@GNR) into A549 cells was confirmed by Raman results (Figure 6); cells were treated with CMO:Eu@GNR (without and with Ab) and irradiated by a NIR laser for 5 min (1 W cm^–2^). Figure 7(A) shows the fluorescence images of phase, live (green), and dead (red) cells with and without NPs treatments. Most of the cells were alive in the control group, whereas a few were dead in CMO:Eu@GNR groups, but complete cell death was observed in the CMO:Eu@GNR-Ab group within the external 808 nm laser exposure area. Moreover, there was no significant difference in the cell viability between no-laser and laser control groups, suggesting that 808 nm NIR laser has a negligible effect on the cells under similar conditions. Besides testing on cancer cells A549, noncancerous hepatocyte AML12 cells were also treated with CMO:Eu@GNR (with and with Ab). As shown in Figure S14, it was also found that the CMO:Eu@GNR have no PTT effect on AML12 cells, suggesting the HNPs with conjugated anti-EGFR antibodies are specific to cancer cells that overexpressed EGFR. The quantitative cell viability of A549 cells without and with treated (CMO:Eu@GNR and CMO:Eu@GNR-Ab) groups for 2 h was studied on 5 min 1 W cm^–2^ 808 nm laser irradiation (Figure 7(B)). The CMO:Eu@GNR group shows a slight toxicity (viability 90 ± 8%), compared to the control group. However, the CMO:Eu@GNR-Ab group shows the least percentage of viability (5 ± 2%), indicating the high PTT effect from the Ab-labeled NPs. The inset of Figure 7(B) shows the fluorescence images of the A549 cells incubated with the CMO:Eu@GNR-Ab NPs where the laser spot edge (white dashed line) shows a boundary between green (no laser) and red (laser) fluorescence regions. Within the laser spot, almost all cells were killed and displayed red color (dead cells). Furthermore, WBC were treated with HNPs to evaluate their biocompatibility. The WBC count demonstrates that HNPs treated groups (HNPs, HNPs-Ab, HNPs + laser, HNPs-Ab + laser) show a slight decrease in count by 9% as compared to the control group (Figure S15). The decrease of WBC count for NP treated groups largely comes from the immune function of WBC to protect cells against foreign invaders (HNPs). However, the numbers of WBC for HNPs treated groups are still under normal range (4500–10,000 cells μl^–1^) after this slight decrease, indicating the good biocompatibility of HNPs.

These results demonstrated that the CMO:Eu@GNR-Ab NPs could effectively and specifically kill A549 cells. This was because the CMO:Eu@GNR-Ab CMO:Eu@GNR can target the A549 cells via the interactions between the Ab and EGFR on the cancer cell surface. Thus, the CMO:Eu@GNR with high specificity and PTT efficiency may be of great importance for cancer treatments and have a potential to apply in clinical cancer therapy.

## 4. Conclusions

In summary, multifunctional HNPs were synthesized for *in vitro* fluorescence imaging, SERS detection, and PTT cancer therapy applications. The HNPs stand out because of their efficient NIR light absorption between 700 and 850 nm and their small size leading to the higher possibility of deeper tumor permeation. Fluorescence images show the fluorescent function of the HNPs with fluorescence at 615 nm (^5^D_0_→^7^F_2_) on excitation ~464 nm. Ab was coated on the surface of the HNPs to enhance cellular uptake. The biomechanical experiments shows that the Young’s modulus of the A549 cells decreased whereas the adhesive force increased with the interactions between the HNPs and cells, and these changes further increased in the group of HNPs combined with Ab (CMO:Eu@GNR-Ab). Raman mapping confirmed the distribution of HNPs around the nucleus and membrane region using SERS characteristic peak of MBA at 1078 cm^−1^, and the EF was found to be ~5.0 × 10^5^. Moreover, these HNPs effectively suppressed A549 cell viability upon 808 nm laser irradiation. However, no significant decrease in cell viability of noncancerous cells (AML12) was observed. The PTT efficiency of CMO:Eu@GNR were found to be 25.6%. These properties of HNPs make them favorable for *in vivo* study in future experiments. Thus, a combination of fluorescence imaging, SERS and NIR photothermal ablation of targeted tumor cells would allow multimodal imaging and PTT *in vivo* for future applications.

## Supplemental data

Supplemental data for this article can be accessed here. http://dx.doi.org/10.1080/14686996.2016.1189797

## Disclosure statement

No potential conflict of interest was reported by the authors.

## Supplementary Material

SM-07-28-16_QF.DOCX
