# Supplementary material for: In vitro biomechanical properties, fluorescence imaging, surface-enhanced Raman spectroscopy, and photothermal therapy evaluation of luminescent functionalized CaMoO4:Eu@Au hybrid nanorods on human lung adenocarcinoma epithelial cells
Source: Sci Technol Adv Mater. 2016 Jul 26;17(1):346–60. doi: 10.1080/14686996.2016.1189797 (PMC5101861; doi:10.1080/14686996.2016.1189797)
Supplement: SM-07-28-16_QF.DOCX [file tsta_a_1189797_sm9737.docx]

**Supporting Information**

*In vitro* biomechanical properties, fluorescence imaging, surface-enhanced Raman spectroscopy, and photothermal therapy evaluation of luminescent functionalized CaMoO_4_:Eu@Au hybrid nanorods on human lung adenocarcinoma epithelial cells

Qifei Li^1^, Abdul K. Parchur^1^, Anhong Zhou^*^

*Department of Biological Engineering, Utah State University, Logan, Utah 84322-4105, USA*

**Corresponding author: Anhong Zhou, Department of Biological Engineering, Utah State University, Logan, Utah 84322-4105, USA, Phone: +1 (435)797-2863, fax: +1 (435)797-1248, email:* [*Anhong.Zhou@usu.edu*](mailto:Anhong.Zhou@usu.edu)*.*

^1^These authors contributed equally to this work as first author.


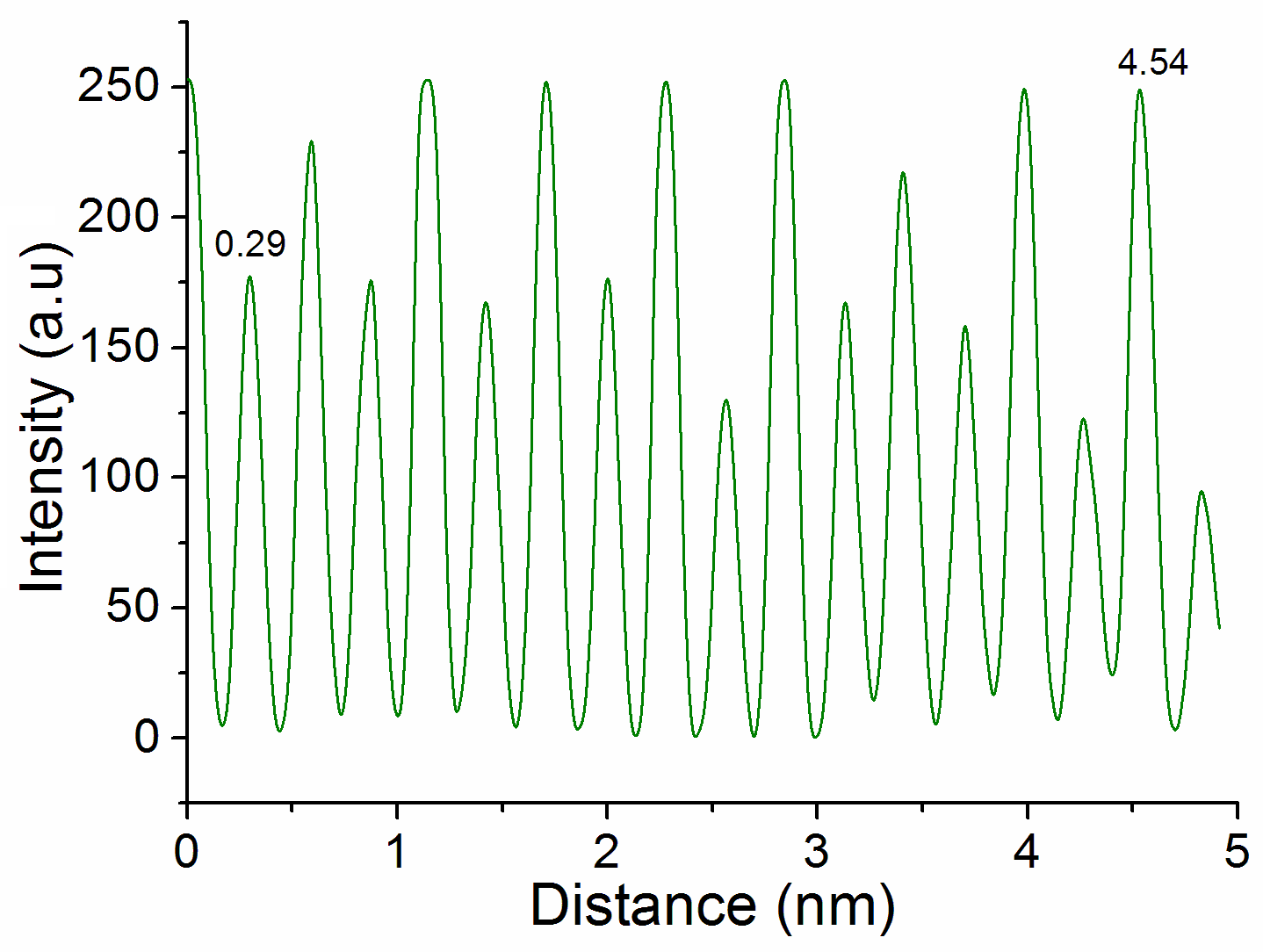


**Figure S1.** Profile plot of the atomic planes of CMO:Eu NPs.


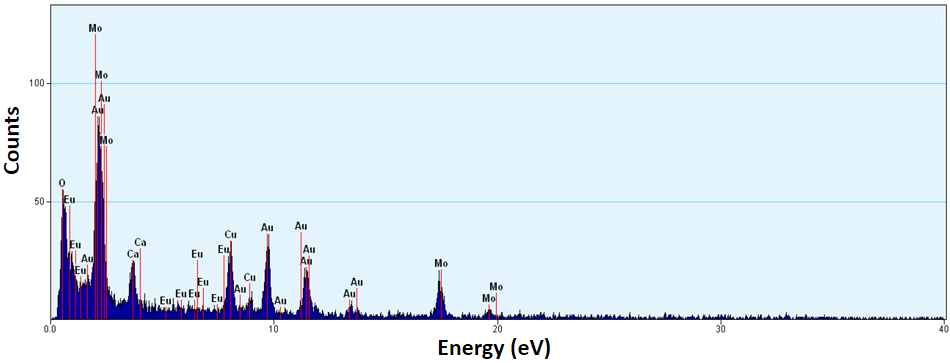


**Figure S2.** Energy-dispersive X-ray spectroscopy measurement profiles of CMO:Eu@GNR HNPs.


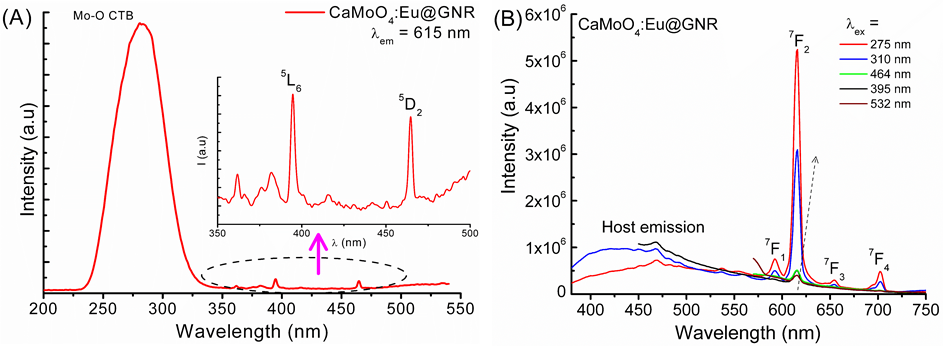
 **Figure S3.** (A) Excitation, and (B) Luminescence spectra of CMO:Eu@GNR HNPs under different excitations.


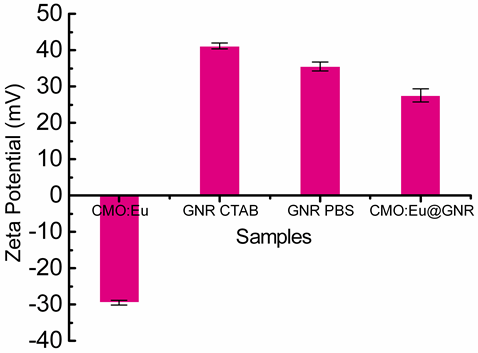


**Figure S4.** Zeta potentials of different samples at pH ~7.

**
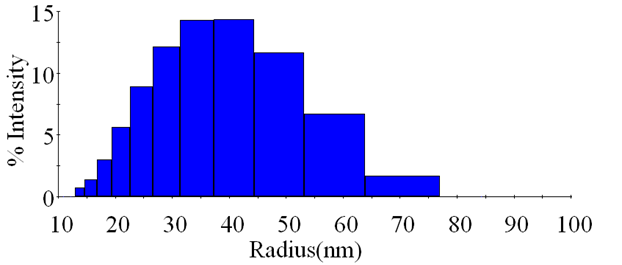
 Figure S5.** DLS data of CMO:Eu@GNR NPs.

**

**

**Figure S6.** Cell viability of A549 cells: control and treated with different concentrations of CMO:Eu@GNR NPs for 24 h (Error bars are standard deviation of the mean, * means P <0.05).


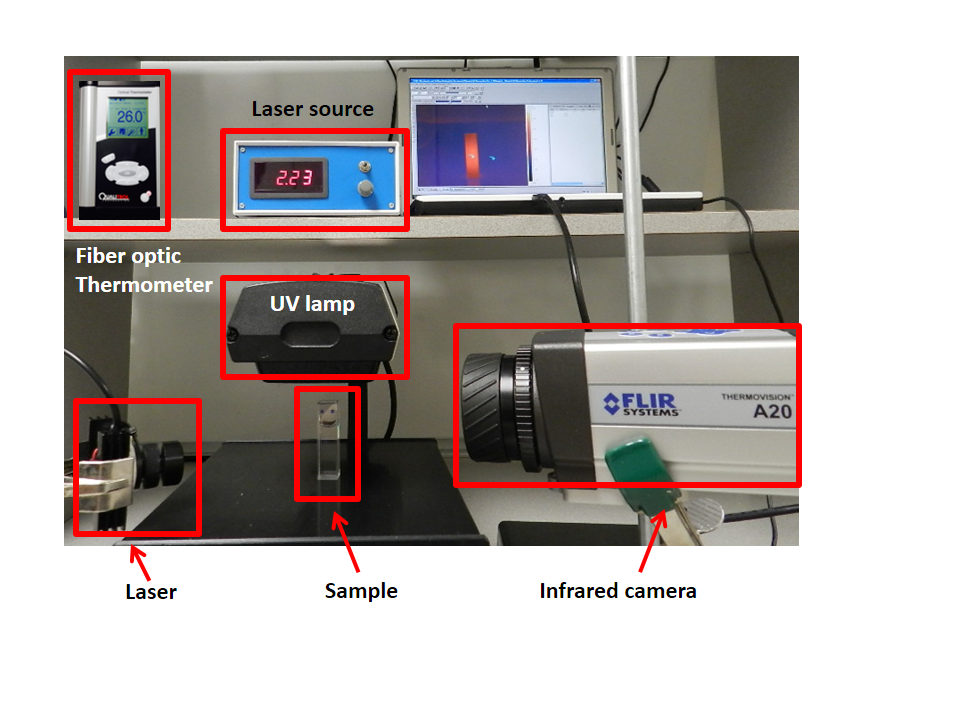


**Figure S7.** Photograph of the photothermal imaging system including an infrared camera, a laser source (808 nm), a UV lamp, and a sample table.


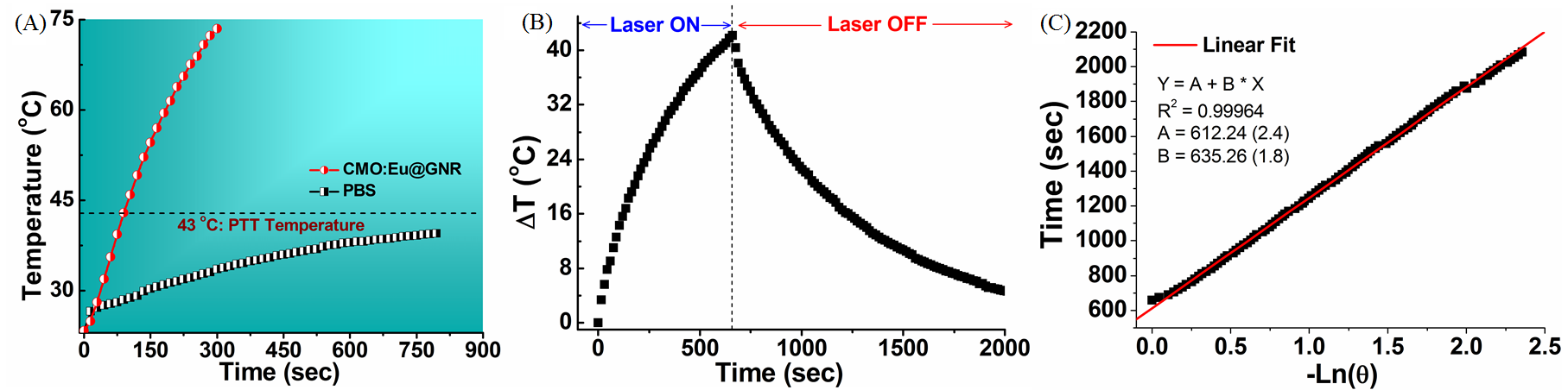


**Figure S8.** Temperature kinetics of PBS and CMO:Eu@GNR samples at 1.5 W/cm^2^ (A). (B) The photothermal response of the CMO:Eu@GNR aqueous solution (28 μg/mL GNR) was recorded for an NIR laser (808 nm, 1.2 W/cm^2^), and then the laser was turned off. (C) The natural log of (T(t) – T_0_)/(T_m_ – T_0_) as a function of the time after the laser was off in Figure (B).





**Figure S9.** Temperature increase of synthesized CMO:Eu@GNR NPs solution as a function of laser power extinction.


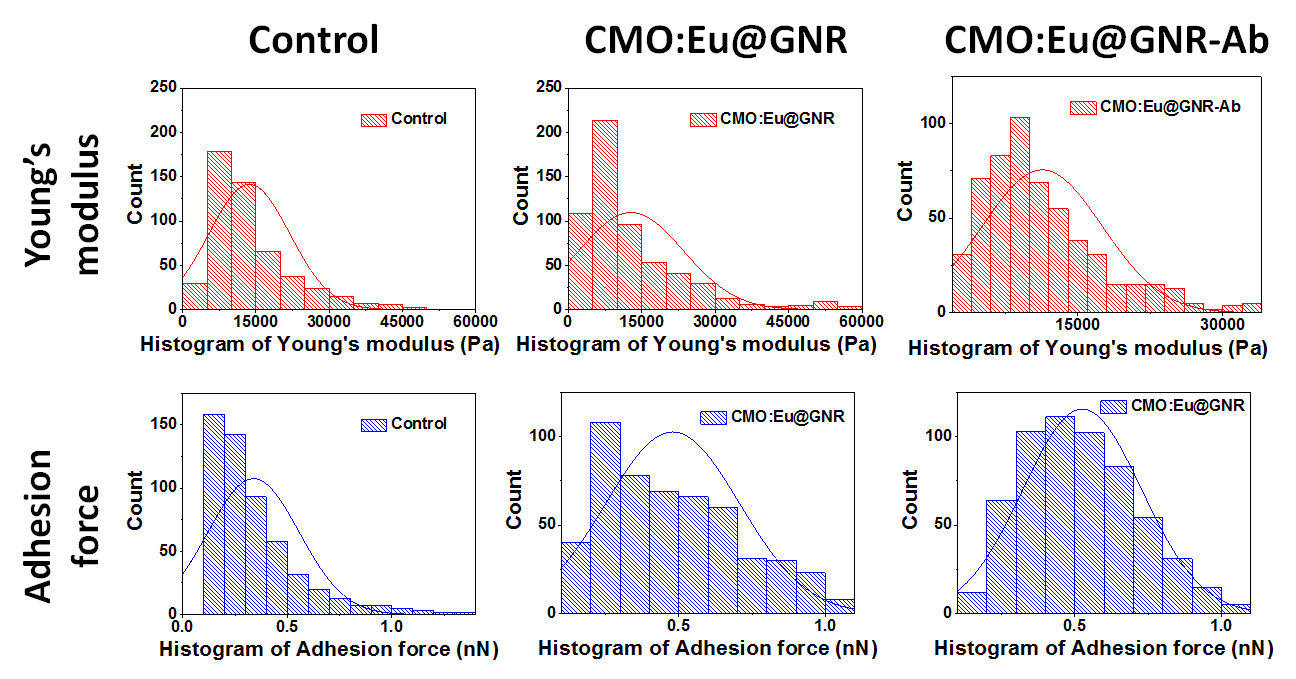


**Figure S10.** Young’s modulus and adhesion force histogram of A549 cells: control and treated with CMO:Eu@GNR and CMO:Eu@GNR-Ab NPs for 2 h.


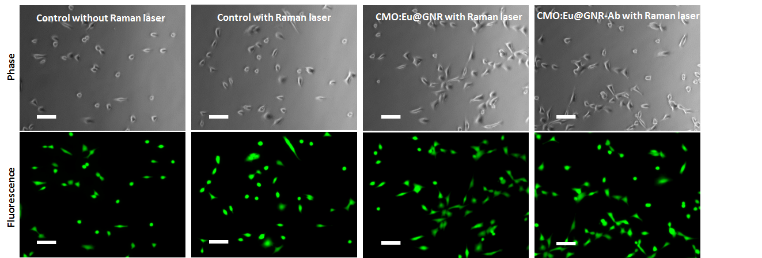


**Figure S11.** Phase and fluorescence images of Calcein AM (green, live cells)/ethidium homodimer-1 (red, dead cells) co-stained A549 cells on MgF_2_ with/without Raman laser and exposed to CMO:Eu@GNR-MBA NPs with and without antibody within 2 h Raman detection. Scale bar: 100 μm.


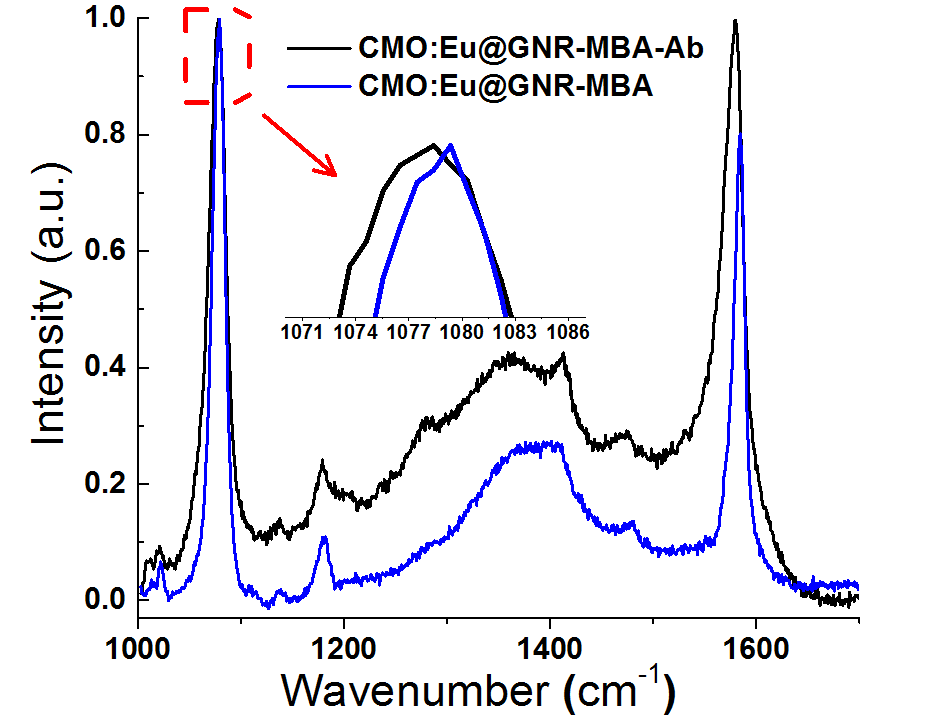


**Figure S12.** Raman spectra of CMO:Eu@GNR-MBA NPs with and without antibody (1078 cm^−1^ selected for normalization).

**
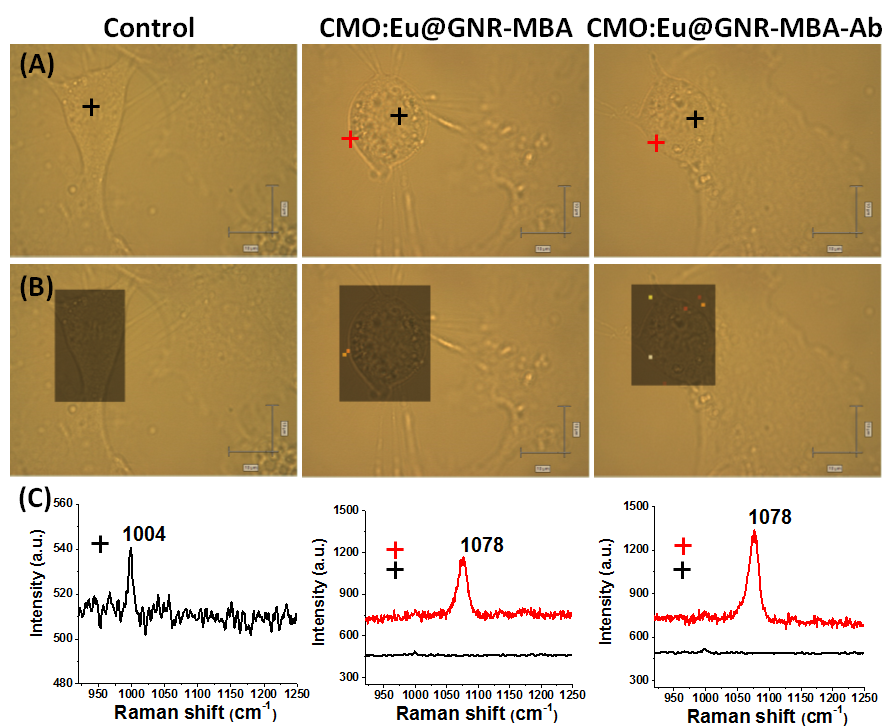
**

**Figure S13.** (A) Raman Bright-field images of AML12 cells without treatment and treated with 2-h CMO:Eu@GNR-MBA or CMO:Eu@GNR-MBA-Ab (peak at 1078 cm^-1^ from MBA was selected for mapping). Scale bar: 10 μm (horizontal), 5 μm (vertical). (B) Raman streamline mapping (Black cross: SERS negative; red cross: SERS positive) and (C) the corresponding Raman spectra of AML12 cells without treatment and treated with 2-h CMO:Eu@GNR-MBA or CMO:Eu@GNR-MBA-Ab NPs at 63× water immersion objective.

*
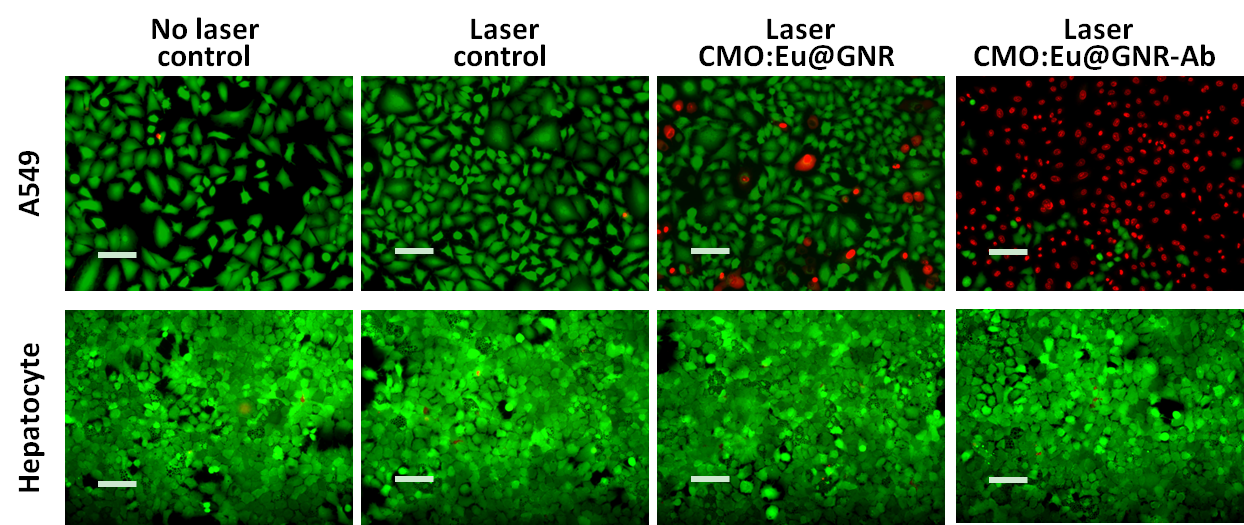
*

**Figure S14.** Fluorescence images of A549 and hepatocyte cells control without laser and with 5 min 1 W/cm^2^ 808 nm laser irradiation and treated with 2 h CMO:Eu@GNR or CMO:Eu@GNR-Ab, then for 5 min 1 W/cm^2^ 808 nm laser irradiation (Green: live cells; red: dead cells. Scale bar: 100 µm).


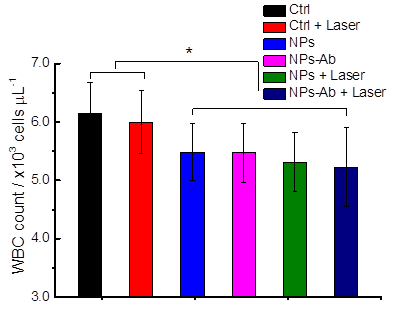


**Figure S15.** The effect of different treatments on the WBC counts (NPs: CMO:Eu@GNR; **p* < 0.05, n=3, error bar: standard deviation of the mean).
